# Yeast-associated skin disease in captive king penguins (*Aptenodytes patagonicus*) caused by an undescribed *Malassezia* species in a Swiss zoological garden

**DOI:** 10.1186/s12917-025-05203-y

**Published:** 2025-12-23

**Authors:** Seraina L. Meister, Sara Soto, Michael Rüttener, Christian Wenker, Sonja Kittl

**Affiliations:** 1 Institute of Animal Pathology, Department of Infectious Diseases and Pathobiology, Vetsuisse Faculty, University of Bern, Länggassstrasse 122, Bern, CH-3012 Switzerland; 2 Zoo Basel, Binningerstrasse 40, Basel, CH-4054 Switzerland; 3 Institute of Veterinary Bacteriology, Department of Infectious Diseases and Pathobiology, Vetsuisse Faculty, University of Bern, Länggassstrasse 122, Bern, CH-3012 Switzerland

**Keywords:** King penguin, Aptenodytes patagonicus, Yeast infection, Fungal feather infection, Malassezia, Staphylococcus kloosii, Opportunistic pathogen

## Abstract

**Background:**

*Malassezia* spp. are lipophilic yeasts that form part of the normal skin flora in many warm-blooded vertebrates but can act as opportunistic pathogens under certain conditions. While commonly implicated in dermatoses in domestic animals, *Malassezia*-associated skin infections in birds, especially aquatic species, are rarely reported. In avian species, diagnostic criteria and treatment protocols are poorly defined, with most knowledge extrapolated from mammals or limited to isolated case reports. Further documentation is needed to better understand host susceptibility, clinical presentation, and management strategies in exotic birds.

**Case presentation:**

Three out of 24 king penguins (*Aptenodytes patagonicus*) housed at Zoo Basel developed bilateral, greasy, light brown deposits on feather tracts at the dorsal and ventral axillary base of the wings and periocular regions. The lesions were non-pruritic and caused no apparent discomfort. Co-housed gentoo penguins (*Pygoscelis papua*) under identical conditions remained unaffected. Histopathology of affected feathers revealed numerous PAS-positive, budding yeast-like organisms measuring 2–3 μm in diameter, consistent with *Malassezia*. Fungal culture and molecular analyses identified a previously undescribed *Malassezia* species, closely related to *M. slooffiae* and *M. gallinae*. A high load of *Staphylococcus kloosii* was also isolated but considered a secondary colonizer. Topical therapy with 0.1% chlorhexidine and 1% terbinafine spray led to clinical improvement in two affected adult penguins. In the third affected penguin, a subadult, oral terbinafine (Terbinafin-Mepha^®^ 250 mg tablets, Mepha-Pharma AG, 16.6 mg/kg SID for 28 days, via medicated feed fish) was initiated due to persistent lesions, poor response to topical therapy, and stress-related intolerance to repeated handling, which also led to anorexia in parts of the colony. However, no clinical improvement was observed. Subsequent treatment with oral itraconazole (Sporanox^®^ 100 mg capsules, Jannsen-Cilag AG; 13.3 mg/kg SID for 28 days, via medicated feed fish) resulted in rapid and complete resolution of clinical lesions in this animal.

**Conclusion:**

This publication expands the host range and clinical spectrum of *Malassezia* skin infections with associated disease to include king penguins. The species-specific occurrence, lesion distribution, and variable therapeutical response highlight the importance of recognizing fungal integumental infections in exotic avian species. It also underscores diagnostic challenges and management considerations, including stress-induced immunosuppression and the need for species-appropriate therapeutic protocols in zoological settings.

## Background

*Malassezia* belonging to the phylum of the *Basidiomycota*, family *Malasseziaceae*, order *Malasseziales* and class *Malasseziomycetes*, are small, thick-walled, ovoid, ellipsoid or cylindrical, lipophilic yeasts that form part of the normal cutaneous and mucosal microbiota in warm-blooded vertebrates, including humans and many animal species. While generally commensal, these organisms can become opportunistic pathogens under specific conditions, such as allergies, keratinization disorders, or immunosuppression [1–6]. *Malassezia*-related dermatitis and otitis externa are commonly encountered in dogs and cats in daily veterinary practice [3, 5, 6]. However, these conditions can also affect farm animals, particularly horses and goats, where their prevalence may be underestimated [7–15]. Additionally, *Malassezia* dermatitis and otitis externa have been reported in a variety of other mammal animal species, including ferrets, okapi, rhinoceroses, and pinnipeds (sea lions and harbor seals) [16–23]. *Malassezia*-associated dermatitis in mammals typically presents as alopecia, erythema, scaling, crusting, and the accumulation of greasy, malodorous, brown to black, kerato-sebaceous debris [14, 15, 24–26]. Chronic infections may also present with lichenification and hyperpigmentation [25]. The intensity of pruritus is variable [4, 6, 12, 14, 15, 19, 24–28]. Across various bird species, different *Malassezia* spp. have been isolated from both healthy and diseased sites, including the beak (*M. brasiliensis*, *M. psittaci*), feathers and wings (*M. pachydermatis*, *M. furfur*), oropharynx (*M. pachydermatis*, *M. furfur*, *M. brasiliensis*, *M. psittaci*), and feces (*M. pachydermatis*, *M. furfur*) [29–33]. *M. sympodialis* has additionally been reported as a component of the resident flora of the chicken combs [34]. Recently, the species *M. gallinae* isolated from chicken skin and ears has been described [35].

Diagnosis of *Malassezia* infection/disease is typically based on clinical examination, cytology, histopathology, fungal culture and molecular techniques like PCR. Treatment protocols are not well established for birds but generally mirror mammalian approaches, involving topical antifungals and, in severe or chronic cases, systemic therapy. Management also includes identifying and addressing underlying predisposing factors.

In this report, the clinical, histopathological, fungal, bacteriological and molecular biological findings of a yeast-infection in three king penguins (*Aptenodytes patagonicus*) from a zoological garden in Switzerland (Zoo Basel) caused by a novel *Malassezia* sp. are described.

### Case presentation

**Fig. 1:**
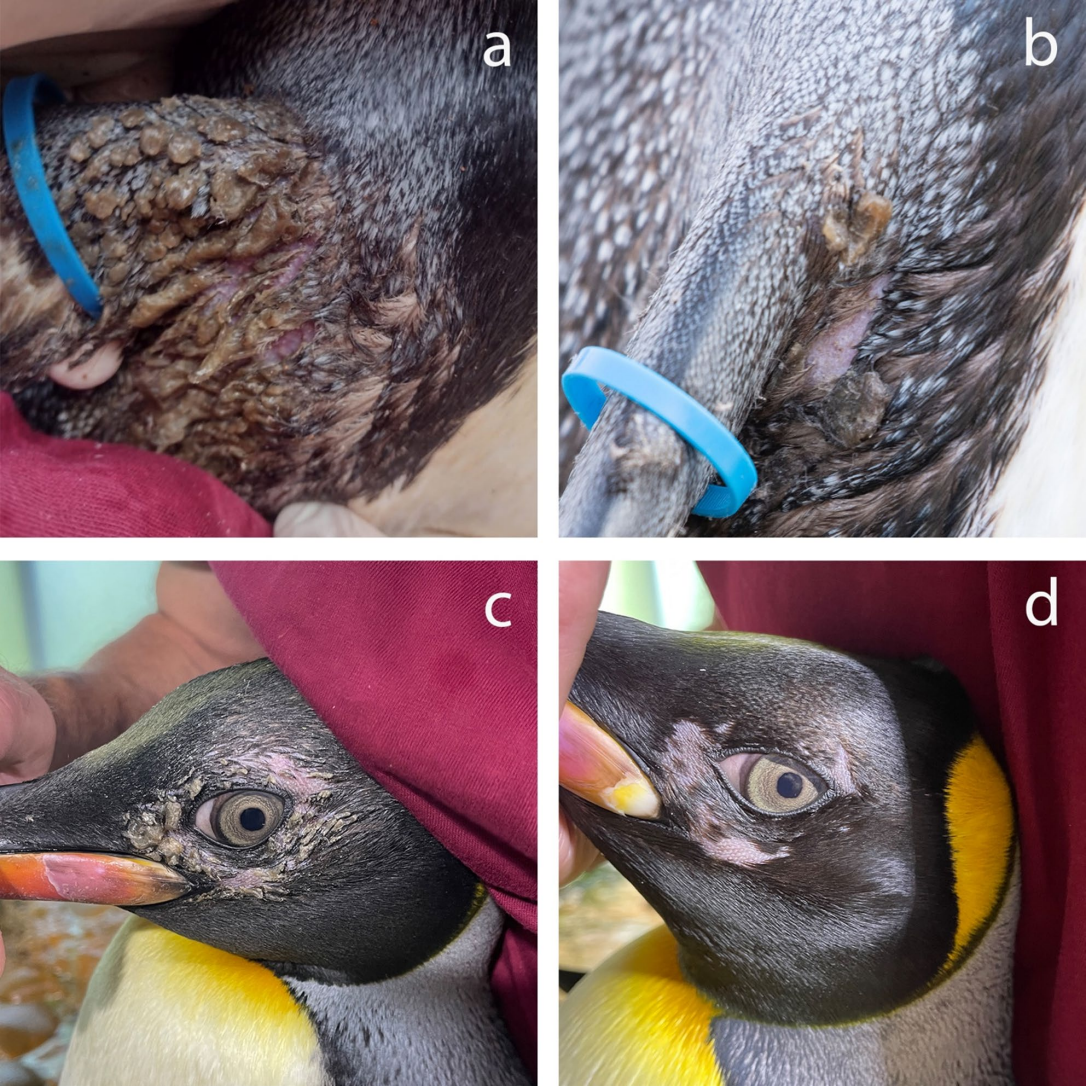
Bilateral, greasy, light brown deposits at the axillary base of the wing of the oldest affected king penguin (**a**, **b**) and the periocular region of the youngest affected individual (**c**, **d**). **a** Before treatment. **b** After 5 days of topical treatment. **c** After unsuccessful topical and oral terbinafine treatment. **d** After 28 days of oral itraconazole treatment.

One subadult (1.5 years old) and two adult (12.5 and 26.5 years old) king penguins (*Aptenodytes patagonicus*) from a group of 24 housed at Zoo Basel presented with bilateral, greasy, light brown deposits located on the feather tracts bilaterally at the dorsal and ventral axillary base of the wings (Fig. 1a and 1b) as well as the periocular region (Fig. 1c and 1d). The deposits were confined to the feathered regions and were not associated with any apparent discomfort or behavioral changes in the birds. Gentoo penguins (*Pygoscelis papua*) maintained under identical husbandry and dietary conditions in the same enclosure were not affected. Both penguin species were housed in an indoor enclosure with a controlled ambient temperature of approximately 8 °C. Water temperatures within the enclosure typically ranged from 8 to 10 °C, with occasional elevations up to 13 °C during peak summer periods. The ambient humidity was generally below 50%, but it could temporarily increase during daily cleaning due to intensive sprinkling with water. During winter months, when ambient outdoor temperatures fell below 10 °C, the penguins were provided access to an outdoor enclosure and routinely taken on supervised walks with zookeepers.

The lesions were identified during daily routine visual check by the animal keepers. Affected individuals were physically restrained for inspection, sample collection, and attempted cleaning of the deposits.

At the time of examination, outdoor temperatures were below freezing, and the deposits were notably hardened, impeding their removal. The material was resistant to cleaning and initial attempts to remove the material using warm water and chlorhexidine solution were only partially successful but was possible to a limited extent by gentle mechanical action using gloved fingernails. The underlying skin appeared grossly mildly erythematous, without evidence of erosion, ulceration, exudation, crusting or similar deposits directly on the skin.

Formalin-fixed and native samples of feathers with deposits were collected for histopathology, fungal and bacterial culture, respectively.

**Fig. 2:**
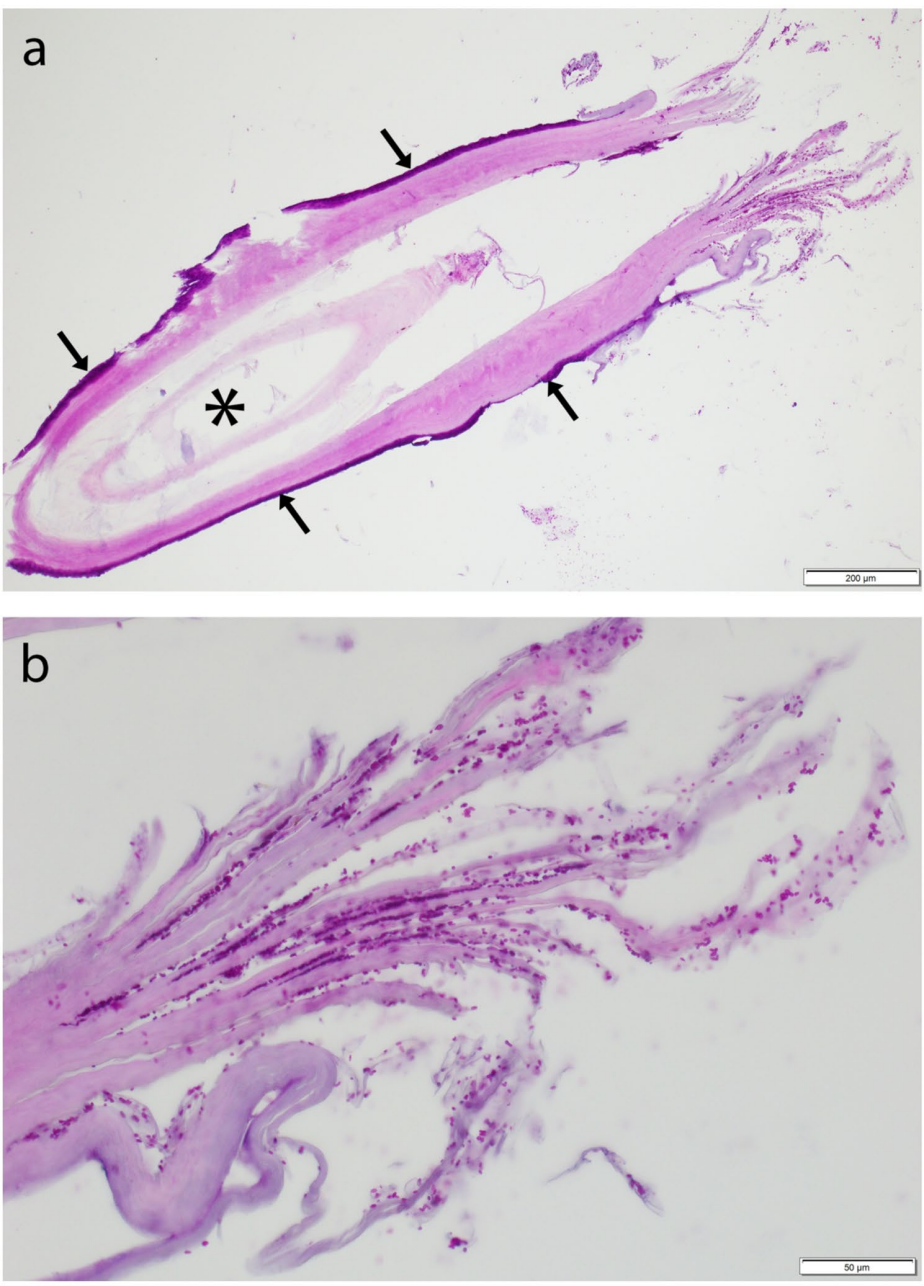
Histopathology of feather samples of the youngest affected king penguin. **a** Overview of an affected feather with feather outer sheath consisting of laminated keratin (arrows), and feather pulp (asterisk), scale bar = 200 μm, PAS-stain. **b** Closer view of the laminated keratin of the feather showed in a, presenting numerous rounded to peanut shaped, 2–3 microns in diameter sized, PAS-positive yeast-like organisms, consistent with *Malassezia*, scale bar = 50 μm, PAS-stain

The pathological examination was performed at the Institute of Animal Pathology, Vetsuisse Faculty, University of Bern. Macroscopic examination of the submitted material revealed 30 feathers with mild deposits of beige, pasty material surrounding the quill. Most feathers (eight, nine and ten feathers per block, respectively) were in toto embedded and processed in paraffin blocks, from which Hematoxylin and eosin (HE) and Periodic acid-Schiff (PAS) stains (with serial sections) were performed. Despite the high number of processed feathers, only scant material was available on the slides for the histopathological examination. It mainly consisted of sloughed lamellar orthokeratotic keratin, with fewer cross-sections from the feathers, which usually presented an incomplete outer epithelial sheath consisting of keratin that appeared split (Fig. 2a). In the sloughed keratin as well as at the surface and inside few feathers there were numerous rounded to peanut shaped, approximately 2–3 microns in diameter sized, yeast-like organisms that presented budding figures and stained positive with PAS stain (Fig. 2b). Multifocally, coccoid bacteria were also present.

Fungal and bacteriological cultures were performed at the Institute of Veterinary Bacteriology, Vetsuisse Faculty, University of Bern. For bacteriological isolation, samples were cultured on sheep blood agar (BD Trypticase™ Soy Agar with 5% sheep blood) and Brolac agar (Axonlab) at 37 °C for 48 h. After two days, a high density of small white colonies developed, which were identified as *Staphylococcus kloosii* using matrix-assisted laser desorption/ionization time-of-flight mass spectrometry (MALDI-TOF MS, Bruker), along with a small amount of mixed flora.

To isolate *Malassezia* spp., samples were cultured on Tween 80 agar (Sabouraud 4% Glucose Agar with chloramphenicol [Roth] supplemented with 0.3% Tween 80 [Merck]) and on Tween 60 olive oil agar (Sabouraud 4% Glucose Agar with chloramphenicol [Roth], 0.3% Tween 60 [Merck], and 0.3% olive oil), then incubated at 37 °C for 7 days.

Moderate growth of small colonies was observed exclusively on Tween 60 olive oil agar (designated isolate 25MD0131). Identification via MALDI-TOF MS was unsuccessful, but Gram staining revealed yeast-like cells consistent with *Malassezia*. Growth was additionally evaluated at 30 °C on Tween 60 olive oil agar, confirming that the strain was capable of growth at this temperature, although colony size was reduced compared to 37 °C.

The internal transcribed spacer 2 (ITS2) region, a universal fungal barcode, was amplified using primers ITS3 and ITS4 and submitted for Sanger sequencing (Microsynth) [36]. BLASTN (NCBI) analysis showed 98% identity with *Malassezia* strains NHC26-1 (PP873993.1) and NHC26-2 (PP873994.1), both previously isolated from chicken ears, followed by *Malassezia slooffiae* CBS 7956^T^ (NR_103584.1) with 94% identity [35]. These strains are phylogenetically close to, but distinct from, *M. gallinae*, a novel species also described in that study, suggesting that they may represent a separate, yet undescribed species [35].

**Fig. 3:**
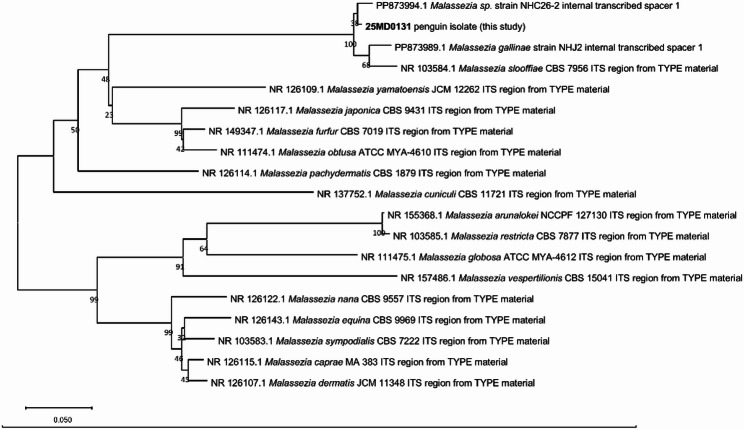
Neighbor-joining tree based on ITS sequences created with MEGA11. The percentage of replicate trees in which the associated taxa clustered together in the bootstrap test (100 replicates) are shown below the branches. The evolutionary distances were computed using the Jukes-Cantor method and are in the units of the number of base substitutions per site. The rate variation among sites was modeled with a gamma distribution (shape parameter = 1). All positions containing gaps and missing data were eliminated (complete deletion option). There was a total of 277 positions in the final dataset. Strain 25MD0131 clusters closely with strain NHC26-2 belonging to a previously undescribed species

For phylogenetic analysis, ITS sequences of type strains were retrieved from GenBank, and a neighbor-joining tree was constructed using MEGA11 [37]. Isolate 25MD0131 clustered with strain NHC26-2 within a broader clade that includes *M. gallinae* and *M. slooffiae* (Fig. 3).

**Fig. 4:**
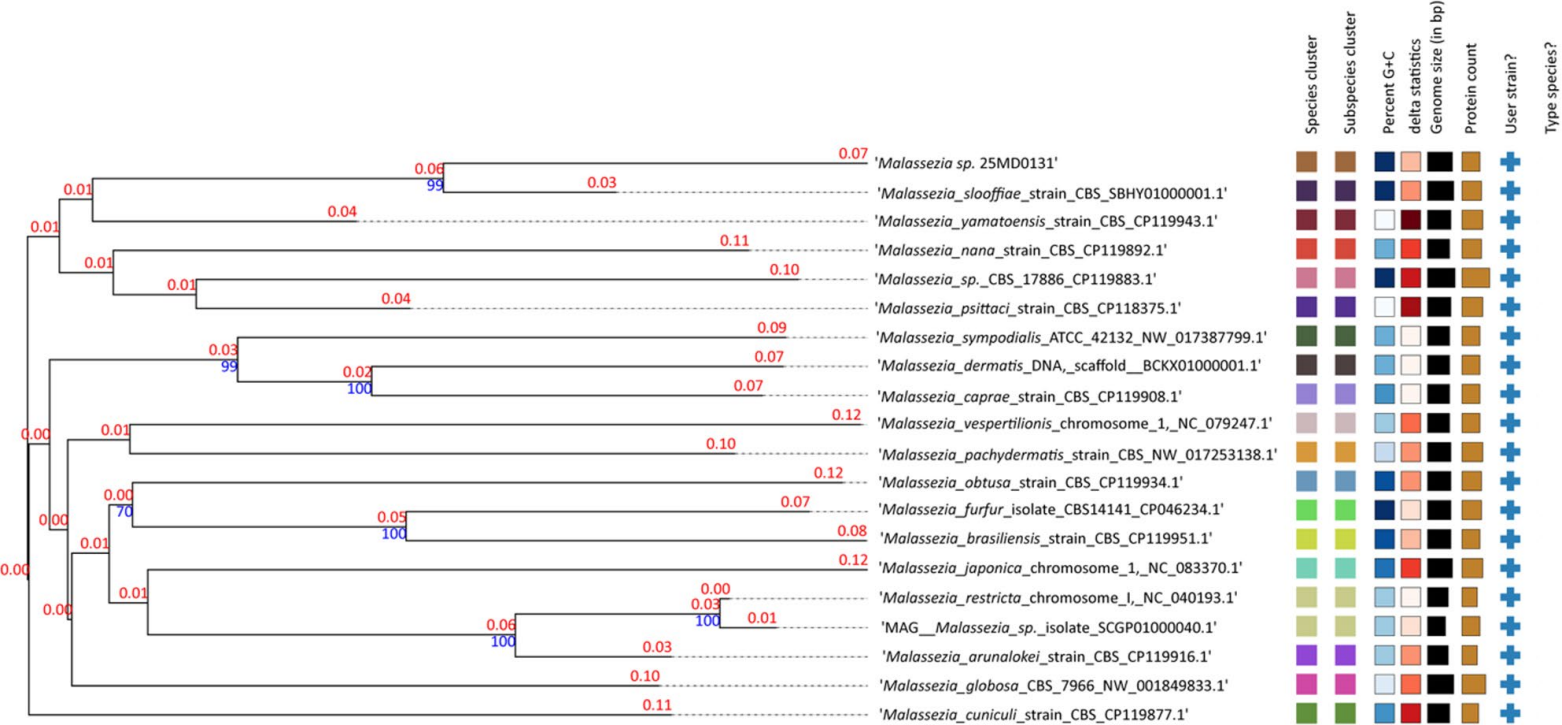
Whole-genome-based phylogeny employing digital DNA-DNA hybridization (dDDH) methods created using the TYGS platform. Isolate 25MD0131, obtained from penguin skin lesions, clusters most closely with *M. slooffiae*, corroborating the ITS-based phylogenetic placement. The colors indicate that the isolates belong to different species. Heatmaps indicate: Percent G + C 49.64–66.74; Delta statistics: 0.361–0.518; Genome size (bp): 6’457’454–9’312’381; Number of Proteins: 5’331–9’274 [39].

**Fig. 5:**
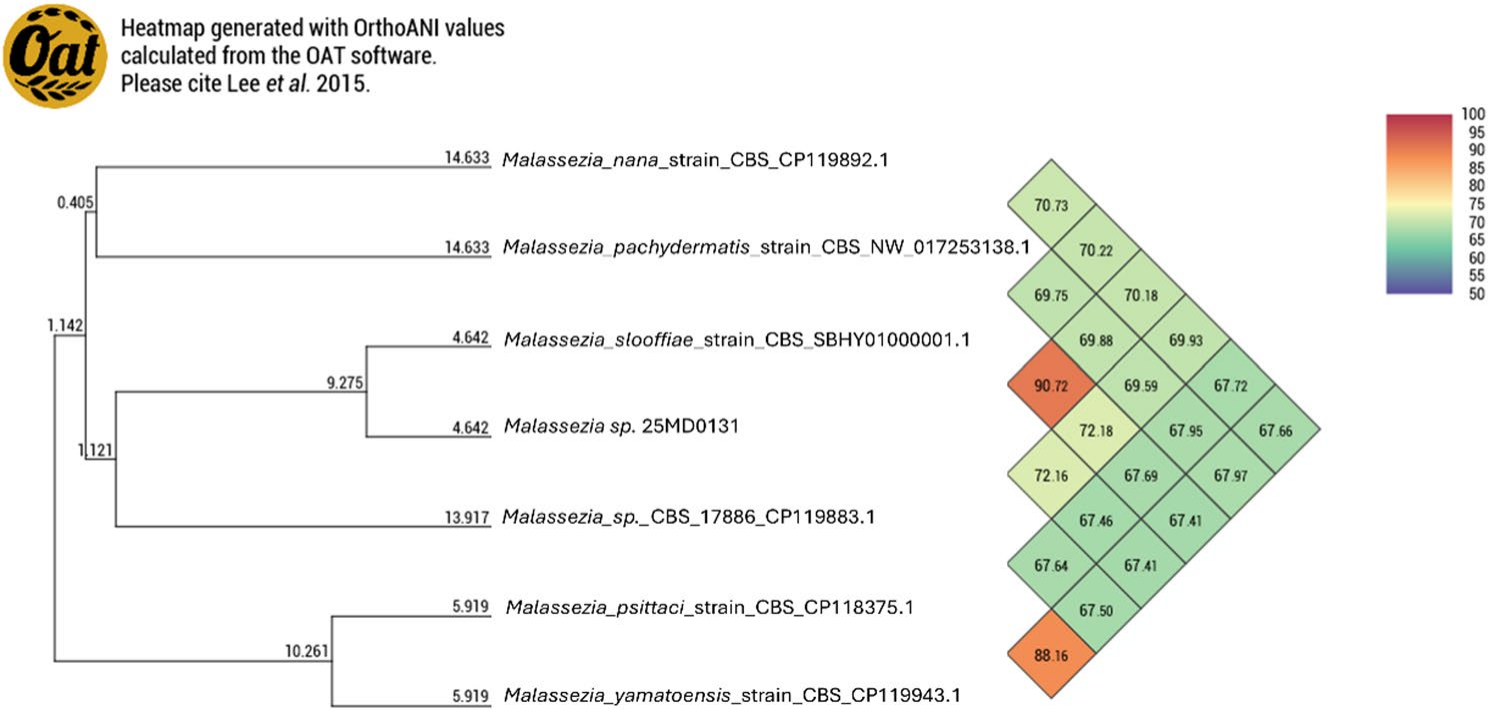
Average nucleotide identity (ANI) between isolate 25MD0131, obtained from penguin skin lesions, and available genomes of the most closely related *Malassezia* species.

For further characterization, genomic DNA was extracted from the isolate and submitted for hybrid Oxford Nanopore Technologies (ONT) and Illumina sequencing (Microsynth). The obtained reads were assembled using Unicycler v0.4.4, and the assembly was cleaned and sorted using Funannotate v1.8.17. The finalized assembly was deposited in GenBank (BioProject: PRJNA1279124, Accession: JBPGNM000000000). For comparative analysis, genomes of other *Malassezia spp*. were downloaded from GenBank (NCBI). Unfortunately, whole-genome sequences for *M. gallinae* and *Malassezia sp.* NHC26 were not available and could therefore not be included in genome-based comparisons. Genomic similarity was assessed using digital DNA-DNA hybridization (dDDH) via the TYGS platform (https://tygs.dsmz.de/) and average nucleotide identity (ANI) analysis using OAT v0.93.1. Although primarily developed for prokaryotes, the commonly used species delineation thresholds of dDDH < 70% and ANI < 95% may also be applicable to yeasts. However, due to limited data, no clear taxonomic consensus exists [38]. Isolate 25MD0131 exhibited an ANI of 90.7% and a dDDH value of 40.6% (based on the recommended d4 formula) in comparison to *M. slooffiae* (Figs. 4 and 5).

Genomic analyses indicate that the penguin isolate 25MD0131 does not correspond to any previously described *Malassezia* species, as the level of genetic divergence exceeds established interspecific thresholds. As a whole-genome sequence for strain NHC26-2 is not yet available, it remains unclear whether this strain represents the same species as the penguin isolate.

As an interim management measure, increased access to indoor swimming was implemented to promote soaking and softening of the feather deposits, facilitating their removal and supporting potential resolution of the condition.

Based on histopathological and microbiological findings, a topical antibacterial and antifungal treatment was prescribed for the three affected animals. This included daily application of 0.1% chlorhexidine solution and 1% terbinafine spray (Lamisil Pedisan^®^ Spray, Medius AG, 10 mg terbinafine hydrochloride per gram) for 40 days. After five days of treatment, a marked reduction in feather deposits was observed, although mild erythema of the underlying skin persisted (Fig. 1b). Treatment was continued as prescribed. One week later, further improvement was noted in the two adult animals, while the subadult showed disease progression of periocular lesions. A follow-up examination one week thereafter revealed marked improvement in all three individuals, and topical therapy was maintained.

Three weeks after initiation of treatment, both handling and topical application were temporarily discontinued for one week due to fish refusal in several individuals of the colony, likely stress related to the manual capture events. Since no deterioration of feather lesions occurred during the treatment pause, therapy was not resumed in the adult penguins. However, the subadult individual still exhibited moderate lesions. As he demonstrated stress-related behavior during handling, systemic therapy with oral terbinafine (Terbinafin-Mepha^®^ 250 mg tablets, Mepha-Pharma AG, 16.6 mg/kg SID for 28 days) was initiated. At a recheck 3.5 weeks later, lesion status in all three animals remained static - no deterioration in the untreated adults and no improvement in the orally treated subadult (Fig. 1c). Therefore, oral therapy was changed to itraconazole (Sporanox^®^ 100 mg capsules, Jannsen-Cilag AG, 13.3 mg/kg SID for 28 days) in the subadult individual which resulted in fast and complete clinical resolution of the greasy deposits in this animal (Fig. 1d). All three affected penguins molted without complication thereafter with no abnormalities in their plumage. Ongoing surveillance has not revealed any further instances of disease among the penguins in the enclosure.

## Discussion and conclusion

This case report describes a novel presentation of *Malassezia* associated skin disease in a small subset of king penguins (*Aptenodytes patagonicus*) in a mixed-species captive colony, with no comparable lesions observed in co-housed gentoo penguins (*Pygoscelis papua*).

The detected *Malassezia* isolate cannot be assigned to any currently recognized species. It likely represents a novel species; however, additional isolates are required to enable a comprehensive characterization and formal description.

It is noteworthy that only king penguins were affected, while gentoo penguins, maintained under identical environmental, dietary, and husbandry conditions, remained clinically unaffected. Neither species is known to be naturally exposed to *Malassezia* spp. in the wild and the selective manifestation of clinical signs in king penguins may therefore reflect species-specific susceptibility, potentially arising from differences in skin physiology, feather microclimate, immune competence or behavioral factors such as grooming. Importantly, the penguin keepers manage multiple enclosures and work with a variety of other avian species, including additional penguins (*Spheniscus demersus*), pelicans, flamingos, various ducks, and kea parrots. Notably, no similar skin lesions were observed in these species, suggesting that the infection was confined to the clinically affected king penguins. Given that the isolated *Malassezia* sp. is closely related to *M. slooffiae*, which has been reported in humans as well as in cats, cattles, sheeps, goats, pigs and horses, potential sources of introduction onto the penguins’ skin warrant consideration [40]. Transmission from humans, such as animal keepers or veterinarians, or from other animals present at the zoo cannot be excluded. Moreover, the potential zoonotic risk of this organism for humans handling the affected animals should be acknowledged, highlighting the importance of strict hygiene and biosecurity measures in the management of captive avian species. The observed lesions were spatially restricted to the periocular area and the axillary feather tracts, which may represent favorable microclimatic conditions regarding temperature, humidity, feather density, or grooming-induced mechanical factors that facilitate yeast overgrowth.

Although *Malassezia* spp. are considered commensal organisms on the skin of many endothermic animals, they can act as opportunistic pathogens under predisposing conditions [3–6]. As noted in the introduction, the pathogenicity of *Malassezia* is often associated with local or systemic immunosuppression [1–6]. In this context, the role of stress-induced immunomodulation in captivity should be considered, as handling, environmental changes, or social dynamics may contribute to localized or systemic immunosuppression in susceptible individuals, thereby facilitating opportunistic fungal infections. Potential risk factors contributing to the development of clinical fungal skin disease include both systemic and local immune impairment. General predisposing conditions may involve environmental or social stress, as well as suboptimal hygiene due to inadequate air or water filtration systems within penguin enclosures. However, no such factors were identified in this case, and the affected individuals had no known prior clinical histories that could explain the observed lesions. Individual or local factors may include pre-existing systemic or dermatological disease or minor skin injuries that could serve as port of entry for bacterial and fungal pathogens. All affected penguins exhibited similar lesions in the axillary region, which is the area where penguins typically rest their beaks during sleep. Sharp and moist beak tips partially covered with fish debris may provide an ideal substrate for a fungal colonization at this site. Furthermore, low ambient temperatures (< 0 °C) were recorded at time the lesions were first detected. Under these conditions, fish debris became frozen within the axillary feathers, potentially impairing normal preening and local hygiene. The exact cause why only three out of 24 king penguins developed lesions despite shared housing conditions remains unclear. This observation suggests a multifactorial etiology, influenced by a combination of host susceptibility, localized environmental factors, individual variability in immune status as well as behavioral or hierarchical stress differences.

Reports of *Malassezia*-associated lesions in birds, particularly aquatic avian species, remain rare, and treatment protocols for these infections in avian species are poorly defined, with limited documentation in the scientific literature [29–33, 36]. Most available information is derived from extrapolation of findings in mammals or isolated case reports in companion birds. Pneumonia and airsacculitis caused by *Aspergillus* spp. are common health concerns in penguins. Consequently, several antifungal agents, including terbinafine and itraconazole, are listed as safe options for off-label use in this species. Different therapeutic approaches were used in the described penguins including application of topical and systemic oral antifungal drugs. Topical treatment using terbinafine was initially selected, primarily due to the availability of this drug in spray form. The use of topical itraconazole formulations would have required shampooing of the affected areas, which was expected to increase stress not only in the affected individuals but also in their co-housed conspecifics. In addition, topical therapy was preferred at first because of its lower risk of systemic adverse effects compared with oral administration. Daily topical application of terbinafine spray (and chlorhexidine spray) resulted in improvement to complete resolution in the two affected adults. However, both the affected penguins as well as the rest of the group exhibited increasing reluctance and anorexia, necessitating discontinuation of the topical treatment. The remaining treatment-resistant, subadult penguin was subsequently treated using oral terbinafine, administered orally via hand-fed fish. After 28 days without clinical improvement, therapy was switched to itraconazole, which proved effective. These outcomes highlight potential variability in drug efficacy and underscore the need for individualized treatment approaches in exotic species.

*Staphylococcus kloosii*, a coagulase-negative staphylococcus (CoNS), was also isolated in high quantities from the bacteriologically examined sample of the affected penguins, likely corresponding to the coccoid bacteria observed histologically. This bacterial species has been isolated from the skin of various wild animals and rarely on that of farm animals and may be regarded as a skin commensal or environmental contaminant in wild animals [37]. Given the histological findings, it is unlikely to be the primary pathogen; however, its abundance along with histological evidence of coccoid bacterial colonization, suggests a role as a secondary colonizer. Additionally, its presence emphasizes the importance of interpreting culture results within the broader clinical and histopathological context for differentiating between true pathogens and contaminants or opportunistic flora in culture-based diagnostics, particularly in integumentary conditions. It is noteworthy, that no concurrent systemic or topical antibiotic treatment was administered. Initially, the penguins received a combination of terbinafine and chlorhexidine sprays. Although chlorhexidine is not an antibiotic in the strict sense, it possesses antibacterial activity. The two adult individuals showed marked improvement to complete healing during this combined therapy, whereas the younger penguin did not respond. Clinical resolution in this individual occurred only after antifungal monotherapy, confirming that the recovery was specifically associated with antifungal rather than antibacterial effects.

This report expands the known host range of *Malassezia*-associated skin disease in aquatic birds and highlights the diagnostic and therapeutic challenges associated with managing fungal infections in zoo-housed birds. In such settings, handling-induced stress and other captivity-associated factors may contribute to immunosuppression, facilitating or exacerbating opportunistic infections and complicating clinical management. These findings further emphasize the need to consider underlying immunological or host-specific predispositions when evaluating the emergence of such infections in novel avian hosts.

In conclusion, the opportunistic behavior of other *Malassezia* species cannot be assumed for this isolate, and its pathogenic potential remains to be confirmed. Investigating its presence on the healthy skin of penguins and assessing host-related predisposing factors would be valuable.

## Data Availability

Data is provided within the manuscript. Sequence data have been deposited in GenBank (BioProject: PRJNA1279124, Accession: JBPGNM000000000).
